# Supplementary material for: Titin-Truncating variants Predispose to Dilated Cardiomyopathy in Diverse Populations
Source: medRxiv. 2024 Jan 17:2024.01.17.24301405. Preprint. [Version 1] doi: 10.1101/2024.01.17.24301405 (PMC10827233; doi:10.1101/2024.01.17.24301405)
Supplement: Supplement 1 [file media-1.pdf]

DATA SUPPLEMENT: Titin-Truncating variants Predispose to Dilated Cardiomyopathy in Diverse Populations

Authors: John DePaolo, MD, PhD,[1] Marc Bornstein, AB,[2] Renae Judy, MS,[1] Sarah Abramowitz, BA,[1] Shefali S. Verma, PhD,[3] Michael G. Levin, MD,[2,4] Zoltan Arany, MD, PhD,[2*] Scott M. Damrauer, MD,[1,2,4,5*]

Affiliations:
[1] Department of Surgery, Perelman School of Medicine, University of Pennsylvania, Philadelphia, PA 19104, USA.
[2] Cardiovascular Institute, Department of Medicine, Perelman School of Medicine, University of Pennsylvania, PA 19104, USA.
[3] Department of Pathology and Laboratory Medicine, Perelman School of Medicine, University of Pennsylvania, PA 19104, USA.
[4] Corporal Michael J. Crescenz VA Medical Center, Philadelphia, PA 19104, USA.
[5] Department of Genetics, Perelman School of Medicine, University of Pennsylvania, Philadelphia, PA 19104, USA.
* Jointly supervised this work

Address for correspondence:
Scott Damrauer, MD
Perelman School of Medicine
Division of Vascular Surgery
Hospital of the University of Pennsylvania
3400 Spruce Street, 14th Floor South Perelman Center
Philadelphia, PA 19104
Office: 215-615-1698
scott.damrauer@pennmedicine.upenn.edu

Tables and Figure Legends

Supplemental Table 1: Number of Individuals Genetically Similar to a 1000 Genomes Project Reference Population in Each Decile of Genetic Distance from the European Centroid.

GD Decile from European Centroid	EUR	AFR	AMR	SAS	EAS
1	4226	42	1	0	0
2	4134	133	2	0	0
3	3814	447	8	0	0
4	3246	1013	10	0	0
5	2579	1679	10	1	0
6	2172	2085	10	2	0
7	2110	2134	21	4	0
8	2476	1734	49	9	0
9	2948	1205	95	20	0
10	2251	664	360	523	470

GD = Genetic Distance; EUR = 1000 Genomes Project European Reference Population; AFR = 1000 Genomes Project African Reference Population; AMR = 1000 Genomes Project Ad Mixed American Reference Population; SAS = 1000 Genomes Project South Asian Reference Population; EAS = 1000 Genomes Project East Asian Reference Population.

Supplemental Table 2: Effect of hiPSI *TTN* truncating variants, genetic distance from 1000 Genomes Project European centroid, and the interaction between genetic distance and hiPSI *TTN* truncating variants on risk of dilated cardiomyopathy.

**Regression Variable**	**Odds Ratio of DCM**	**95% Confidence Interval**	***P*-value**
hiPSI TTNtv	5.63	4.16 to 7.61	<0.001
GD	7.84×10^{-8}	1.63×10^{-16} to 23.30	0.10
hiPSI TTNtv*GD	1.84×10^{24}	4.09×10^{-9} to 8.27×10^{56}	0.15

hiPSI = high percentage spliced in; DCM = Dilated cardiomyopathy; GD = Genetic Distance from the 1000 Genomes Project European Centroid.

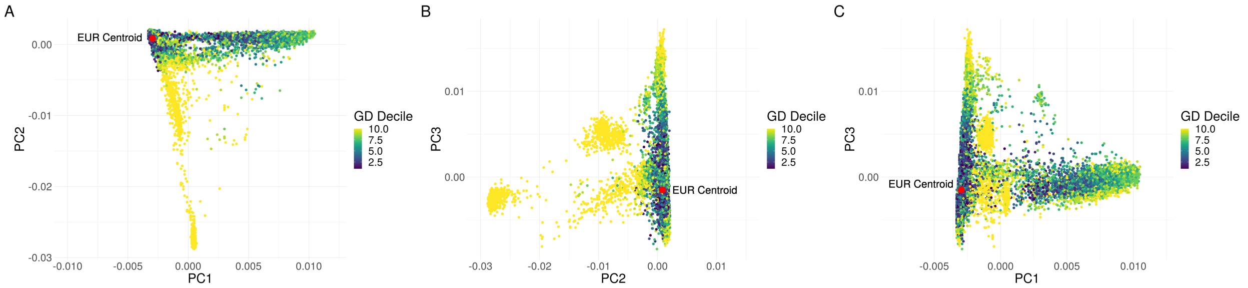

Supplemental Figure 1: Principal component based clusters of individuals in the Penn Medicine Biobank compared to genetic distance from the 1000 Genomes Project European centroid. Discrete labelling of the position of each individual within PMBB colored by decile of genetic distance from the 1000 Genomes Project (1000G) European centroid in a plot of (**A**) principal component (PC) 1 versus PC2, (**B**) PC2 versus PC3, and (**C**) PC1 versus PC3 with 1000G European centroid (red dot) identified in each plot demonstrating the overlap between individuals genetically close or distant from the 1000G European centroid based on PC space.

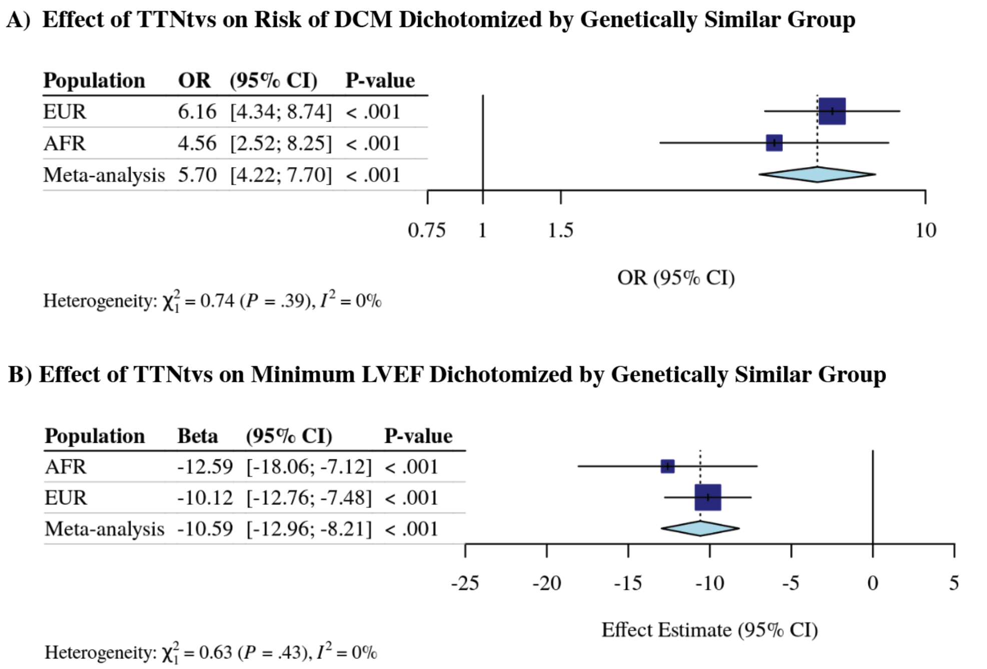

Supplemental Figure 2: Effect of high percentage spliced in titin truncating variant on risk of dilated cardiomyopathy diagnosis and minimum left ventricular ejection fraction reduction stratified by genetically similar group without excluding those with ischemic cardiomyopathy in the Penn Medicine Biobank. (**A**) Logistic regression analysis of the association between hiPSI TTNtvs and DCM diagnosis, and (**B**) linear regression analysis of the association between hiPSI TTNtvs and minimum left ventricular ejection fraction among individuals genetically similar to the 1000 Genomes Project European and African reference population, and meta-analyzed. OR = odds ratio; CI = confidence interval; EUR = individuals genetically similar to the European reference population; AFR = individuals genetically similar to the African reference population.

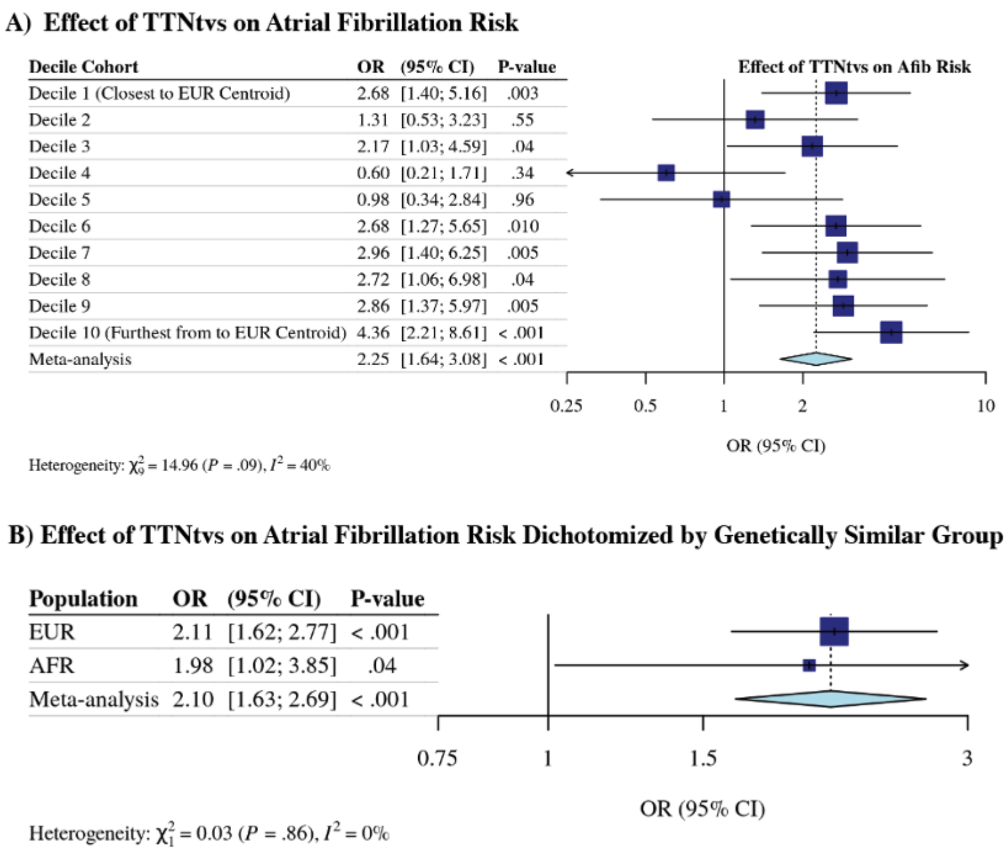

Supplemental Figure 3: Effect of high percentage spliced in titin truncating variant on risk of atrial fibrillation diagnosis in the Penn Medicine Biobank. (**A**) Logistic regression analysis of the association between hiPSI TTNtvs and Afib by deciles of genetic distance from the 1000 Genomes Project European centroid; and (**B**) logistic regression analysis of the association between hiPSI TTNtvs and Afib by genetically similar group. OR = odds ratio; CI = confidence interval; EUR = individuals genetically similar to the European reference population; AFR = individuals genetically similar to the African reference population.
